# Understanding Pain and Quality of Life in Paediatric Cancer Survivors: A Systematic Review with a Focus on Early Survivorship

**DOI:** 10.3390/children12101320

**Published:** 2025-10-02

**Authors:** Francesca Di Domenico, Christina Liossi, Sandrine Martine Géranton

**Affiliations:** 1 Department of Cell and Developmental Biology, University College London, Gower Street, London WC1E 6BT, UK; sandrine.geranton@ucl.ac.uk; 2 School of Psychology, University of Southampton, Highfield Campus, Southampton SO17 1BJ, UK; c.liossi@soton.ac.uk

**Keywords:** children, pain, cancer, survival, anxiety, depression, fatigue

## Abstract

**Highlights:**

**What is already known on this topic?**

- Children and young people (CYP) who survive cancer—particularly females—are more likely than the general population to report acute and chronic pain in later life, and they frequently experience comorbidities such as fatigue, depression, and anxiety. Yet, their pain is often likely to be under-recognized, especially in childhood, partly due to a lack of developmentally sensitive assessment tools in CYP.

**What this study adds?**

- This study demonstrates that the poor understanding of post-cancer treatment pain in children and young people (CYP) stems not only from the absence of adapted assessment tools but also from limited early post-treatment data. Together, these shortcomings create a significant gap in mapping CYP pain trajectories after cancer.

**How might this study affect research, practice or policy?**

- Our findings highlight the urgent need for timely, rigorous, age-specific research to better inform survivorship care and shape evidence-based clinical policy. Our study also suggests that clinicians should routinely assess both acute and chronic pain in CYP cancer survivors using validated, age-appropriate tools in early post-treatment completion assessments.

**Abstract:**

Background: Pain is a common but often under-recognized clinical feature among children and young people (CYP) cancer survivors. This systematic review aimed to examine the prevalence of acute and chronic pain in 5–24-year-old cancer survivors, explore associated biopsychosocial comorbidities and evaluate the psychometric properties of pain assessment tools used with this population. Methods: This review provides a conceptual replication to a review published in 2020 by Schulte et al. by updating and purposefully narrowing the review population to CYP (5–24 y) to better capture their pain experience. We updated the search from Schulte et al., extending the search period up to October 2024 across 5 databases. Results: Our independent search identified 18 studies, with only 1 new paper published since Schulte et al., 2020. Overall, CYP cancer survivors reported a higher prevalence of pain than the general population, with females experiencing higher levels than males, consistent with broader chronic pain literature. Fatigue, depression, and anxiety were common comorbidities, and pain substantially impacted quality of life. Key limitations included the use of unvalidated questionnaires; inconsistent definitions of chronic pain and lack of patient stratification based on diagnoses, age and treatment protocol. Moreover, data were aggregated, and we were unable to extract information from early survivorship. Conclusions: There is a critical need for more rigorous research on pain in CYP cancer survivors. Specifically, gathering data on pain experienced during the immediate post-treatment completion phase of cancer care, an area currently underrepresented in the literature, will provide valuable insights into patients’ pain trajectories.

## 1. Introduction

Survival rates for most paediatric cancers have improved dramatically over the past four decades [1,2]. As the number of paediatric cancer survivors is growing, it is essential to prioritize the enhancement of their quality of life, short-term and long-term. For this, it is necessary to fully understand the challenges survivors face beyond treatment, including the prevalence, type, impact and management of pain throughout survivorship. Pain is indeed a key factor that significantly affects CYP survivors’ quality of life, psychosocial health, and overall well-being [3]. The pain reported is often recurring, chronic (lasting more than 3 months), complex and varied, including neuropathic pain from nerve damage, musculoskeletal pain linked to growth or mobility changes, and headaches or abdominal pain of unclear origin [4].

While pain management is a critical but under-studied outcome of cancer treatment, only a few studies have been published focusing on cancer-related pain in CYP [5,6,7,8]. Indeed, according to the World Health Organisation’s “Guidelines on the management of chronic pain in children” published in 2020: (I) “systematic reviews of effectiveness did not identify any studies in children less than 10 years of age”; and (II) “research is needed for children with chronic cancer-related pain during or following cancer treatment”. Children with cancer frequently report pain, starting from the moment of the diagnosis to the end of life or to survivorship [9,10]. The pain is certainly the result of a variety of processes, from the cancer itself to the medical interventions used for its management, including chemotherapy and radiotherapy [9]. Other important factors to consider when examining cancer-related pain in CYP include the psychological trauma associated with the diagnosis of a potentially life-threatening condition and the impact of numerous hospital visits and interactions with the medical staff. These experiences likely contribute to the overall pain experience, as stress alone is expected to exacerbate pain in adolescents, as observed in adults [11,12]. Pain in CYP is a complex biopsychosocial experience that significantly impacts the quality of life of both individuals and their families [13]. It is conceptualized through the interaction of biological, psychological, and social factors within a dynamic cognitive framework [14]. Interdisciplinary interventions provide the most effective management approach [13,15,16,17].

The most recent systematic review of 73 studies by Schulte et al. [18], which we conceptually replicated, showed that childhood cancer survivors are at high risk of experiencing pain, with pain being more prevalent in females and commonly associated with fatigue, emotional distress, anxiety and depression. Although Schulte et al. [18] provided important insights into pain in paediatric cancer survivors, significant gaps in the evidence base remained, which limited our understanding of pain experiences in this population. Their review, consistent with the available literature, seldom examined pain as a primary outcome and was constrained by studies that often-lacked validated assessment tools, clearly defined pain measures, and attention to age-, diagnosis-, and treatment-specific differences. Moreover, age groups were frequently combined, offering no information on the immediate post-treatment completion phase, where early pain trajectories and intervention opportunities may be particularly critical.

Addressing these gaps is essential to develop targeted, evidence-based interventions that improve long-term quality of life for children and young people (CYP) survivors. The present conceptual replication of the Schulte et al. [18] review therefore aimed to (a) assess the prevalence and characteristics of acute and chronic pain in CYP cancer survivors (up to 24 years), with a specific focus on the early post-treatment period; (b) evaluate the methods used to assess pain and their psychometric properties; (c) identify biopsychosocial outcomes associated with pain, and its impact on quality of life; (d) provide recommendations for future research and clinical practice.

## 2. Materials and Methods

In October 2020, Schulte F. et al. [18] conducted a systematic review examining the prevalence of pain in paediatric cancer survivors; the current review conceptually replicates their work by focusing on a narrower population, replicating and updating the literature searches up to October 2024 and conducting more rigorous assessments of the risk of bias of included studies and additional assessments of the psychometric properties of the questionnaires used in included studies. A conceptual replication is a “purposeful broadening or narrowing of the research question in existing systematic reviews” (e.g., across broader or more focused populations, intervention types, settings, outcomes, or study designs) and as such it is realistic to view the two reviews as overlapping systematic reviews rather than truly replicated or duplicate systematic reviews [19].

The present systematic review was conducted in accordance with Cochrane guidelines [20] and reported using the Preferred Reporting Items for Systematic Reviews and Meta-Analyses (PRISMA) standards [21]. The review includes studies investigating acute or chronic cancer-related pain in survivors of any type of paediatric cancer. Although adolescence has traditionally been defined as ending at 18 years, more recent perspectives recognize that the transition from childhood to adulthood extends beyond this age. Sawyer et al. [22] argue that defining adolescence as ages 10 to 24 more accurately reflects developmental processes and societal norms. In line with this contemporary view, we have adopted an adolescent age range extending to 24 years for the purposes of this review. Screening of the articles and data extraction were conducted independently by 2 reviewers, incorporating both newly identified studies and those from the original review. The review protocol was prospectively registered with PROSPERO (registration ID CRD420250622420).

### 2.1. Databases Searched

Databases searched included PubMed (Web-based), PsycINFO (EBSCO), EMBASE (Ovid), EMBASE (SCOPUS) and Web of Science (Thomson Reuters). Full PubMed search parameters are available (Table S1). Search strategies for additional websites were customized to each database’s syntax, using a combination of thesaurus terms and keywords. Relevant articles from the original review and new ones between October 2020 and October 2024 were included. Only original research articles were considered, though narrative and systematic reviews were assessed to identify relevant original studies. Dissertations, books, book chapters, editorials, letters, case studies, and conference proceedings/abstracts were excluded.

Ethical approval was not required for this study, as it is a systematic review of previously published literature and does not involve any primary data collection involving human participants or animals.

Patient and Public Involvement: No patient involved.

### 2.2. Endpoints

The primary endpoint of this review was the prevalence of acute and chronic pain in children and young people (CYP; 5–24 years) surviving cancer. Secondary endpoints included (i) characteristics of acute and chronic pain, biopsychosocial comorbidities associated with pain (e.g., fatigue, depression, anxiety), (ii) the impact of pain on quality of life, and (iii) the psychometric properties of pain assessment tools applied in this population.

### 2.3. Inclusion Criteria

The inclusion criteria were defined as follows: (1) original research, (2) assessment of pain included, (3) children diagnosed with cancer between the ages of 0 and 21, and (4) survivors aged between 2 and 24 who were at least 5 years post-diagnosis and/or 2 years post-completion of therapy. Eligibility criteria based on the PICOS framework can be found in Table 1. 

### 2.4. Risk of Bias

Risk of bias was assessed for each study using an adapted ROBINS-I tool [23], which was developed to evaluate non-randomized intervention studies. We conducted independent quality assessments based on five key criteria: (1) confounding, (2) participant selection, (3) intervention classification, (4) missing data, and (5) outcome measurement. While the original ROBINS-I framework uses a five-point scale (low, moderate, serious, critical, no information), we simplified this to three levels (low, moderate, high) to improve clarity and consistency. This adaptation was necessary because the papers lacked sufficient methodological detail for more granular assessment. None of the studies reached the “critical” threshold, making the distinction between “serious” and “critical” risk uninformative for our synthesis. We classified “no information” as high risk to acknowledge the uncertainty introduced by missing methodological details. This three-tier classification approach is commonly used in systematic reviews [24].

## 3. Results

### 3.1. Data Extraction

**Figure 1:**
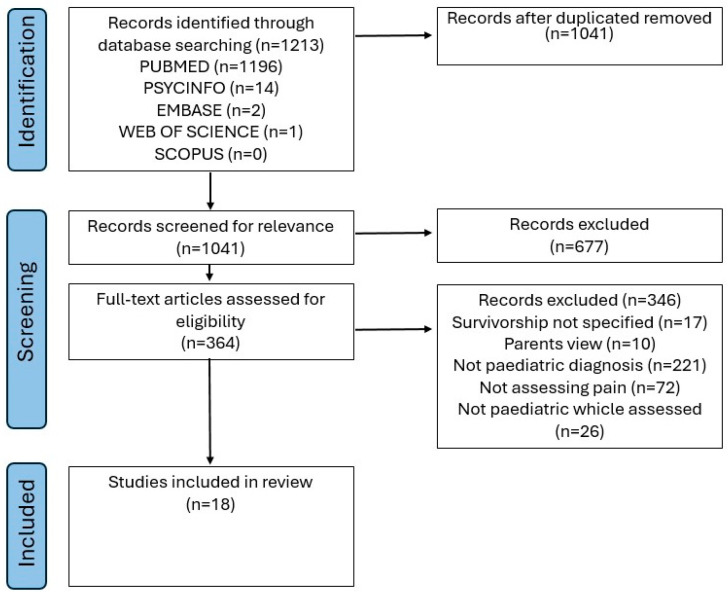
PRISMA chart.

The review identified 1213 unique publications based on title and abstract screening, of which 18 articles were included in the final review (Figure 1). Any disagreements were resolved through consensus.

### 3.2. Quality Assessment

The most frequent source of potential bias was confounding: 11 of 18 studies (61%) were rated at high risk, with none judged at low risk. For participant selection, 6 studies (33%) were rated high risk and 11 (61%) low risk. Intervention classification was another area of concern, with 15 studies (83%) rated high risk and only 3 (17%) low risk. In the missing data domain, most studies (17/18, 94%) were judged to have moderate risk of bias, with one study rated low risk. For outcome measurement, 6 studies (33%) were rated high risk and 11 (61%) low risk. Detailed GRADE assessment can be found in Table 2.

### 3.3. Data Synthesis

#### 3.3.1. Characteristics of Included Studies

The majority of studies were observational, cross-sectional studies (77.77% *n* = 14) or cohort (16.66% *n* = 3), with the remainder classified as non-experimental (1% *n* = 1). Of the 18 assessed studies, 33.33% (*n* = 6) included a comparison group: siblings (*n* = 1), parents (*n* = 1), general population (*n* = 2) other cancer survivors (e.g., survivors with or without chronic pain, *n* = 2). The remaining 66.67% (*n* = 12) did not include a comparison group. Sample sizes varied widely in different studies, ranging from 19 to 1123 participants, and the follow-up period spanned an average of 2 to 15.2 years post-diagnosis. The average age at diagnosis ranged from 3.9 to 19 years old, while the average age at the time of the study ranged from 5 to 24 years old. It was impossible to stratify the data according to post-treatment time because relevant time-point information was not collected or not reported. Among the studies, only 4 (22.22%) identified pain assessment as a one of the primary objectives for the study. Table S2 in Supplementary Data (summary of included studies) presents the descriptive characteristics of the included studies.

#### 3.3.2. Methods for Measuring Pain

The most commonly used measures of pain were validated psychometric questionnaires used by 77.77% of the studies, while items created by authors were used for 27.70% of the studies. One study (5.5%) used an Algometer, a validated measurement of pain tolerance [35] (Table 3). The most common validated surveys used in the studies were Health Utilities Index and Short Form Health Survey (SF-36). Specific approaches for measuring pain are reported into Table S3 in Supplementary Data (Psychometric domains). Of the included studies, five (5/18) investigated chronic pain, with only 3/5 including a definition of chronic pain, defined as pain lasting more than 12 weeks [4], or chronic headache, defined as headaches that occurs 15 or more days per month for at least three consecutive months, whilst the other 2 referred to chronic pain without offering a clear definition. Among these 5 studies, one study focused exclusively on chronic pain, while the remaining four examined both acute and chronic pain. Overall, ambiguity in pain classification was frequent.

### 3.4. Pain Prevalence

#### 3.4.1. Chronic Pain

Only 5 studies (27.77%) investigated chronic pain. All five studies that investigated chronic pain had pain or/and headache assessment as one of their primary objectives. Chronic pain prevalence ranged from 9.5% to 55.0% among survivors. Further, 3 of those 5 studies focused on survivors of acute lymphoblastic leukemia (ALL) [27,32,40], while the others on acute myeloid leukemia neuroblastoma sarcoma, Wilms tumour, kidney tumour and soft tissue sarcoma [26,42]. The five studies reporting chronic pain focused on back pain in ALL survivors [27,32], cancer-related pain, neuropathic pain [26,32,42] migraine and headaches [32,40]. Of these eight studies, only one had a comparison group, such as healthy siblings [27].

#### 3.4.2. Acute Pain

Acute pain, or any other pain not defined as chronic, was reported by 13 studies (72.22%). In addition, four studies reported both acute and chronic pain. Of those 17 studies, only 7 studies (41.2%) identified pain as one of their primary objectives. Pain prevalence ranged from 7.0% to 44.4% among survivors. ALL was the most prevalent form of cancer, reported in 8 of the 17 studies [27,30,32,34,35,38,39,40] followed by leukemia reported in 5 studies [25,31,38,39,41], CNS tumour, lymphoma, Hodgkin’s lymphoma, Wilm’s tumour, sarcoma [28,31], neuroblastoma [37], posterior fossa astrocytoma [36], glioma, germ cell [33] and craniopharyngioma [29]. The 17 studies reporting acute pain focused on bone pain, back pain, and headaches. A comparison group was reported in 6 of the 17 studies (35.3%), including healthy siblings (*n* = 1) [27], general population (*n* = 2) [28,30,38], parents (*n* = 1) [38], and other cancer survivors (*n* = 2) [33,40]. Of those, evidence suggested that survivors of childhood cancer are at higher risk of experiencing pain. Studies show that cancer childhood survivors report pain 4 times more than healthy controls [41], specifically back pain, where 44.4% or survivors reported back pain, compared to 21.1% of siblings [28].

#### 3.4.3. Treatment-Related Pain

A limited number of papers reported treatment-related acute or chronic pain. Children who survived ALL and were treated with a high-risk protocol reported significantly more pain compared to those treated with a standard-risk protocol [31]. Additionally, survivors of acute myeloid leukemia (AML) who underwent chemotherapy followed by allogeneic bone marrow transplant (alloBMT) reported a higher prevalence of chronic health conditions, including pain, at 76%, compared to 58% among those treated with chemotherapy alone or chemotherapy followed by autologous bone marrow transplant (autoBMT) [34]. Another study found that radiotherapy increased the incidence of back pain in children who survived ALL [27].

### 3.5. Pain Correlates

#### 3.5.1. Biological

Female survivors consistently reported a higher prevalence of pain compared to male survivors. Two studies looked at the difference between males and females, and one study evidenced that 60.5% of female ALL survivors reported back pain compared to 32.1% of male survivors; of those, 60.5% of female ALL survivors reported back pain compared with only 14.3% of female siblings. In comparison, 32.1% of male ALL survivors reported back pain compared with 31.8% of male siblings [27]. The other study evidenced that 54% of females reported headaches compared to 41% in male’s survivors [40].

Another study that clustered symptoms among childhood cancer survivors found that, within the neurological symptom cluster, which included back pain and other types of pain, females reported pain significantly more often (96.8%) compared to males (67.2%) [42].

#### 3.5.2. Psychological

The psychological factors explored in the literature included sleep, fatigue, and emotional distress. Fatigue, daytime sleepiness, and difficulty performing daily activities were commonly reported in a limited number of studies [31,42]. Arpaci et al. found that 52% of participants experienced fatigue, with 36% reporting that it limited their ability to work [25]. Another study reported fatigue in 21.6% of patients, with 1.8% experiencing severe fatigue [32]. Fatigue was also found to be associated with moderate to severe headache-related disability [40].

Pain was associated with anxiety [31,34,42], depression [41,42], and suicidal ideation [37]. Cognitive difficulties were also noted in several studies: one reported that 48% of respondents experienced cognitive issues that impacted their educational activities, while another found that 4.1% of survivors reported cognitive impairments [37].

#### 3.5.3. Quality of Life

Quality of life was assessed using validated questionnaires such as the Medical Outcomes Survey Short Form-36 (SF-36) and the 15-item Health Utilities Index (HUI).

Khan et al. found that quality of life was negatively affected by migraines, tension-type headaches, and the presence of back pain in CYP [32]. Portwine et al. reported no significant differences in quality of life based on gender, relapse history, or current disease status when analyzing the HUI-2 (which measures emotional distress and anxiety) and HUI-3 (which assesses emotional well-being and happiness) [37].

Psychosexual functioning was evaluated in one study, which found that 18.4% of survivors felt their illness had limited their sexual life. The same study also reported no differences in quality of life between survivors treated during childhood and those treated during adolescence. However, when compared to the general population, survivors reported lower quality of life in areas such as general health, physical functioning, and social functioning [38].

## 4. Discussion

This review set out to address persistent gaps in the evidence on pain in children and young people (CYP) cancer survivors, with a particular focus on the early post-treatment completion phase, when pain trajectories and intervention opportunities may be most critical. By concentrating on studies that included survivors up to 24 years, we aimed to extend the insights of Schulte et al. [18], whose review was constrained by mixed age groups, infrequent use of pain as a primary outcome, and limited reporting of early survivorship data. Despite this targeted approach, it was not possible to stratify findings by post-treatment time, as relevant information was rarely collected or reported, underscoring the need for more rigorous, methodologically consistent research in this area. Nonetheless, overall, our review confirms the findings from Schulte et al. highlighting several key observations and ongoing gaps in the literature.

Our findings reinforce that both acute and chronic pain are prevalent issues among CYP cancer survivors, with survivors consistently reporting higher rates of pain than control populations when comparison data were available. Female survivors also tended to report higher levels of pain than males, a trend consistent with findings in the broader chronic pain literature beyond oncology [43]. However, many studies lacked control groups, as identified by Schulte et al. [18], potentially limiting the ability to draw robust conclusions. In both Schulte et al. [18] and this review, only a third of studies included a comparison group, including healthy or population controls, siblings, and other cancer survivors. Nonetheless, when comparisons were made, they consistently showed higher levels of pain among survivors, underscoring the added burden associated with childhood cancer. Broader population-level data reinforce this picture: Chambers et al. reported an overall pain prevalence of 20.8% in the CYP population, whereas our findings indicate that pain affects up to 55% of the survivor population [44]. Beyond methodological issues, the more fundamental problem for CYP survivors is that no control group can adequately reflect the cumulative trauma, treatment intensity, and life disruption faced by survivors. As such, it may be impossible to identify a truly equivalent comparator, and alternative strategies—such as longitudinal within-cohort designs—are likely to provide more meaningful insights into pain trajectories and risk factors unique to CYP cancer survivors.

Importantly, pain in CYP survivors was also frequently associated with a range of biopsychosocial comorbidities, including fatigue, depression, and anxiety. These findings underscore the complex and multidimensional nature of pain, which cannot be fully understood or addressed without considering psychological and functional outcomes [45]. In line with this, several studies reported a significant negative impact of pain on quality of life, particularly in domains such as physical functioning, social engagement, and emotional well-being.

A notable limitation across the included studies was the variability and, in many cases, the lack of rigour in pain assessment methods. While most studies used validated questionnaires, a substantial proportion relied on author-created or unvalidated measures, compromising reliability and comparability. Only a few studies employed standardized psychophysical assessments, such as quantitative sensory testing, to quantify pain sensitivity or tolerance a methodology that has provided valuable insights into different phenotypes of a number of paediatric chronic pain conditions [46,47]. Furthermore, many studies did not report the psychometric properties of the tools for paediatric populations, leaving uncertainty about their appropriateness. These methodological limitations restrict confidence in the reported prevalence and characteristics of pain and impede identification of meaningful associations with biopsychosocial outcomes. Future research should prioritize validated, age-appropriate instruments with well-documented psychometric properties to ensure accurate and clinically meaningful measurement of pain in CYP cancer survivors.

Beyond assessment methods, the studies included had several additional limitations. First, pain was inconsistently defined across studies, with acute and chronic pain often grouped together or not clearly distinguished. Moreover, pain was rarely identified as a primary outcome in the studies examined, highlighting a broader issue in survivorship research: the underrepresentation of pain as a clinical priority. Another key limitation was the aggregation of different cancer diagnoses, coupled with the lack of consideration for treatment era. As cancer therapies have evolved over time, generally becoming less toxic in more recent years, the prevalence and nature of pain among survivors may differ across treatment cohorts. Failing to account for cancer diagnosis and treatment differences limits our ability to understand how cancer types and treatment protocols influence long-term pain outcomes.

Finally, only a few studies focused exclusively on paediatric populations, while most combined children and adolescents with young adults. This practice limits the ability to draw age-specific insights and may obscure important developmental differences in pain experience and reporting. Moreover, our study is the first to report a critical gap in the literature: the absence of data on pain in children and young people in the immediate post-treatment completion phase of cancer care. While most studies focus on long-term survivors (often several years post-treatment) and provide valuable insights into late effects, few, if any, address the immediate survivorship phase or stratify the collected data by post-treatment time. However, understanding pain experiences in the early post-treatment period is essential for mapping the trajectory of pain in these children, from its onset to potential progression into late chronic stages. Early pain reports are especially valuable as they more accurately reflect children’s and adolescents’ experiences during cancer therapy, unlike later reports that may be affected by memory biases or adaptation over time [48]. In addition, evidence on the influence of age on pain reports is inconsistent, further reinforcing the need for not-only early but also age-stratified data collection [18]. Collecting data from CYP immediately after treatment would bridge this knowledge gap and provide insights into pain trajectories, ultimately informing the development of targeted early interventions for prevention and management.

In conclusion, this systematic review assessed the prevalence and characteristics of acute and chronic pain in CYP cancer survivors (up to 24 years). Across studies, pain prevalence was high, with chronic pain reported in up to 55% of survivors, yet very few studies treated pain as a primary outcome or collected data during the immediate post-treatment phase, limiting insight into early pain trajectories. In evaluating methods of pain assessment, we found inconsistent use of validated, psychometrically sound tools, with many studies relying on author-created items or unstandardized measures. This variability hinders comparability across studies and highlights the urgent need for validated age-appropriate patient-reported outcome measures (PROMS) and psychophysical assessments tailored to paediatric populations [47]. Our synthesis also identified biopsychosocial correlates of pain. Female survivors consistently reported higher pain prevalence than males, and pain was strongly associated with fatigue, anxiety, depression, and lower quality of life. These findings underline the multidimensional burden of pain and its significant impact on survivors’ physical, emotional, and social functioning. Based on these findings, future research should prioritize rigorous, child-focused study designs that incorporate standardized pain outcomes, validated measures, and stratification by age, diagnosis, treatment type, and survivorship stage. Clinically, routine monitoring of both acute and chronic pain, particularly in the immediate post-treatment completion phase, could identify survivors at risk for persistent pain and enable timely multidisciplinary interventions. Such efforts are essential to improve long-term quality of life for CYP cancer survivors.

## Figures and Tables

**Table 1:** Eligibility criteria based on the PICOS framework. Inclusion and exclusion criteria were defined using the PICOS (Population, Intervention, Comparator, Outcomes, Study design) framework to guide study selection.

Element	Inclusion Criteria	Exclusion Criteria
Population (P)	Children diagnosed with cancer between the ages of 0 and 21 years; survivors aged 2–24 years who were at least 5 years post-diagnosis and/or 2 years post-completion of therapy.	Children diagnosed after the age of 21; survivors older than 24 years.
Intervention (I)	All cancer-related treatments.	Studies not within 5 years post-diagnosis and/or 2 years post-completion of therapy.
Comparator (C)	Studies with and without a comparison group.	None.
Outcomes (O)	Acute and chronic pain, quality of life, and psychological outcomes.	Studies in which pain was not assessed at all.
Study design (S)	Randomized controlled trials and cohort studies.	Reviews, editorials, conference abstracts, dissertations, books, book chapters, letters, and case studies.

**Table 2:** Risk of bias. Risk of bias was evaluated using an adaptation of ROBINS-I across five domains: confounding, participant selection, intervention classification, missing data, and outcome measurement. Each domain was judged as low (green), moderate (yellow) or high (red) risk of bias.

Study	Confounding	Participant Selection	Intervention Classification	Missing Data	Outcome Measurement
Arpaci T. [25]	X	+	-	-	X
Berg C. [26]	-	+	X	-	+
Bowers DC. [27]	X	+	X	+	+
Brinkman TM. [28]	X	X	X	-	+
Crom DB. [29]	-	X	X	-	+
Feeny D. [30]	X	-	-	-	+
Hsiao CC. [31]	X	X	X	-	+
Khan BR. [32]	-	+	X	-	+
Kranick SM. [33]	X	+	-	-	X
Schultz KAP. [34]	-	+	X	-	X
Lieber S. [35]	-	+	X	-	-
Odame I. [36]	-	+	X	-	+
Portwine C. [37]	-	+	X	-	+
Van Dijk EM. [38]	X	X	X	-	X
Revel-Vilk S. [39]	X	X	X	-	X
Sadighi Z. [40]	X	+	X	-	+
Schwartz LA. [41]	X	+	X	-	+
Williamson LR. [42]	X	X	X	-	X

Judgement: X: High; -: Moderate; +: Low.

**Table 3:** Frequency of various approaches used to assess pain and quality of life in reviewed studies.

Health-related quality of life or health status measures	14 (77.77%)
Valid pain measures	1 (5.5%)
Chart review	1 (5.5%)
Author-created measures	5 (27.7%)

## Data Availability

Data from this study are available from the corresponding author upon request.

## Supplementary Materials

The following supporting information can be downloaded at https://www.mdpi.com/article/10.3390/children12101320/s1, Table S1: Systematic Review Search Strategy; Table S2: Summary of the studies included; Table S3: Psychometric domains.
