# Supplementary material for: Understanding Pain and Quality of Life in Paediatric Cancer Survivors: A Systematic Review with a Focus on Early Survivorship
Source: Children (Basel). 2025 Oct 2;12(10):1320. doi: 10.3390/children12101320 (PMC12564031; doi:10.3390/children12101320)
Supplement: Supplementary file 1 [file children-12-01320-s001.zip › children-3845709-supplementary.pdf]

Supplementary tables for: Pain and quality of life in paediatric survivors of childhood cancer: A conceptual replication of the Schulte et al., 2020 systematic review.

Table S1: Systematic Review Search Strategy

1.	neoplasms[mh] OR neoplas*[tiab] OR cancer*[tw] OR tumor*[tiab] OR tumour*[tiab] OR carcinoma*[tiab] OR malignan*[tiab] OR oncolog*[tiab] OR oncolog*[jour] OR metasta*[tiab] OR leukemia*[tiab] OR lymphoma*[tiab] OR “brain cancer”[tiab] OR (brain[ti] AND (cancer*[ti] OR neoplasm*[ti])) OR brain tumor*[tiab] OR brain tumour*[tiab] OR “posterior fossa syndrome”[tiab]
2.	“antineoplastic agents”[pharmacological action] OR "antineoplastic agents"[Majr] OR chemotherapy[tw] OR antineoplastic*[tiab] OR “antimetabolites, antineoplastic”[Pharmacological Action] OR “antimetabolites, antineoplastic”[majr] OR therapy[sh] OR therapy[tw] OR therapeutic[ti] OR treatment*[ti] OR dexamethasone[tiab] OR prednisone[tiab] OR antimetabolite*[tiab] OR methotrexate[tiab] OR cytarabine[tiab] OR “radiotherapy”[majr] OR radiotherapy[tiab] OR radiotherapy[sh] OR radiation[majr] OR radiation[tiab] OR irradiation[tiab] OR “proton therapy”[tiab] OR “neurosurgical

	procedures"[majr] OR neurosurg*[ti] OR anticancer[tiab] OR "anti-cancer"[tiab] OR cerebrospinal fluid shunt*[tw] OR ventriculostomy[tw] OR ventriculoperitoneal shunt*[tw] OR craniotomy[tiab] OR craniectomy[tiab] OR intrathecal[ti]
3.	child[mh] OR child[ti] OR children*[ti] OR kids[ti] OR youth[ti] OR juvenile[ti] OR pediatric*[ti] OR paediatric*[ti] OR infant[mh] OR infant*[ti] OR infancy[ti] OR schoolchildren[ti] OR childhood[ti] OR preschooler*[ti] OR girls[ti] OR boys[ti] OR adolescen*[ti] OR adolescent[mh] OR teen[ti] OR teens[ti] OR teenager*[ti]
4.	"pain"[mh] OR pain*[ti] OR "chronic pain"[tiab] OR "persistent pain"[tiab] OR "episodic pain"[tiab] OR headache*[ti] OR neuralgia[ti] OR myalgia[ti] OR arthralgia[ti]

Table S2: Summary of the studies included

Paper	Study Design	Sampl e Size	Median Age at Diagnosis (years)	Median Age at Study (years)	Median Time Since Diagnosis /Treatmen t (years)	Number of Controls	Pain Outcomes Assessed	Pain Outcome Findings
Arpaci T. et al.	Observationa l – Cross Sectional	91	Mean 6.38	Mean 11.66	Mean time following treatment	N/A	Items developed by authors	Widespread muscle, bone and joint pain

Paper	Study Design	Sample Size	Median Age at Diagnosis (years)	Median Age at Study (years)	Median Time Since Diagnosis /Treatment (years)	Number of Controls	Pain Outcomes Assessed	Pain Outcome Findings
					completion = 2.55			(41.8%). burning and pain during urination reported by 19.8%, more in females than males. Abdominal pain reported by 8.8% and Chest pain reported by 3.3%. The treatment duration of those who have pain is longer than the others who did not have pain (2.99 vs 2.44).
Berg C. et al.	Observational – Cross Sectional	42	Mean 9.8	Mean 20.5	Mean 11.6	N/A	Pediatric Cancer Quality of Life Inventory	18 (43%) participants reported pain, of those 11 (61%)

Paper	Study Design	Sample Size	Median Age at Diagnosis (years)	Median Age at Study (years)	Median Time Since Diagnosis /Treatment (years)	Number of Controls	Pain Outcomes Assessed	Pain Outcome Findings
								indicated that pain limited work activities and 11 (61%) that affected sleep.
Bowers D.C.	Observational – Cross Sectional	99	Not reported but before 18 years	Mean 18.1 (participants) Mean 17.6 (controls)	Not reported, but 5-year survivor	53 siblings	Items developed by the authors	Forty-four (44.4%) survivors reported experiencing back pain compared to 11 (21.2%) of their siblings. 26 (26.3%) survivors reported experiencing back pain of longer than 6-months compared to 6 (12%) of their siblings. 10 (10.1%) survivors reported experiencing

Paper	Study Design	Sample Size	Median Age at Diagnosis (years)	Median Age at Study (years)	Median Time Since Diagnosis /Treatment (years)	Number of Controls	Pain Outcomes Assessed	Pain Outcome Findings
								hip pain versus 1 (2.0%) of their siblings. 26 (60.5%) of female survivors reported back pain versus 18 (32%) of male survivors
Brinkman T. M	Observational – Cohort	1123	Mean 2.6	Mean 15	Mean 12.4	2770	Behavior Problems Index	Cancer-related pain and migraines or severe headaches compared with no pain were associated with increased risk of comorbid symptoms.
Crom D.B.	Observational – Cross-Sectional	51	Mean 7.1	Not specified but mean 7.6	Mean 7.6	N/A	European Organization for the Research and Treatment of	20 survivors (39.2%) reported headache.

Paper	Study Design	Sample Size	Median Age at Diagnosis (years)	Median Age at Study (years)	Median Time Since Diagnosis /Treatment (years)	Number of Controls	Pain Outcomes Assessed	Pain Outcome Findings
							Cancer (EORTC QLQ-C30) – Pain subscale	
Fenny D	Observational – Cross Sectional	69	Mean 5.96	Mean 15.5	Not reported, but 5-year survivor	N/A	Health Utilities Index (HUI) – Pain item	One-fourth of the patients reported multiple health sequelae. 95.7% of participants reported being 'free of pain and discomfort., while 4.3% of patients reported having 'occasional pain.
Hsiao C.C	Qualitative and observational study	201	Not specified but between 0 and 6	Mean 15.5	Mean 7.5	Parent 201	Intensity of treatment rating 2.0	Only 59 survivors (29.4%) didn't experience any adverse health events, and 25 survivors (12.4%)

Paper	Study Design	Sample Size	Median Age at Diagnosis (years)	Median Age at Study (years)	Median Time Since Diagnosis /Treatment (years)	Number of Controls	Pain Outcomes Assessed	Pain Outcome Findings
								experienced five or more adverse health events.
Khan B.R.	Observational – Cross Sectional	162	Mean 3.9	Mean 15.7	7.4 years since last treatment	N/A	Migraine Disability Assessment Scale; Modified Hanover; Low Back Pain Disability Questionnaire; 36-Item Short Form Health Survey (SF-36) – Bodily Pain scale	Migraine headaches were present in 51 (31 %) and 25 (15 %) experienced auras. Tension type headache was diagnosed in 49 (30 %). Both migraine and episodic tension type headaches were present in 24 (15 %) and 18 (11 %) participants had chronic daily headaches. Any neuropathy

Paper	Study Design	Sample Size	Median Age at Diagnosis (years)	Median Age at Study (years)	Median Time Since Diagnosis /Treatment (years)	Number of Controls	Pain Outcomes Assessed	Pain Outcome Findings
								was present in 102 (62.9 %) participants. Glove and stocking type sensory neuropathy was present in 65 (40 %). Autonomic symptoms by questionnaire were present in 47 (29 %) and cranial nerve dysfunction in 10 (6 %) participants. Recurrent back pain was reported by 37 (23 %) participants
Kranick S.M.	Observational – Cohort	265	10.8 years for children without headaches	Not specified, maxiumum 21 years old	Not specified	N/A	Chart review	In 37 patients with recurrent headache, AIS or TIA

Paper	Study Design	Sample Size	Median Age at Diagnosis (years)	Median Age at Study (years)	Median Time Since Diagnosis /Treatment (years)	Number of Controls	Pain Outcomes Assessed	Pain Outcome Findings
			; 8.4 years for children with headaches					occurred in 7 (19%) subjects, compared to 6 patients without headache.
Lieber S.	Observational – Cross-Sectional	48	Mean 5.7	Mean 9.8	Mean 3.2	N/A	Reduced Pediatric-Modified Total Neuropathy Score (rPed-mTNS)/ QST	Five patients (11%) indicated pain by answering the question to the current status “Do you have any parts of your body that are tingly, numb (can hardly feel), or hurt?” with yes. Two patients (4%) reported pain extending to ankles or wrist, 2 (4%) complained

Paper	Study Design	Sample Size	Median Age at Diagnosis (years)	Median Age at Study (years)	Median Time Since Diagnosis /Treatment (years)	Number of Controls	Pain Outcomes Assessed	Pain Outcome Findings
								about pain extending to knee or elbow and one patient reported pain extending above knee or elbow. Sensory NCV was reduced in 7 of 46 survivors (15%) including one reporting pain in rPed-mTNS and two with pathologically high rPed-mTNS. Decreased mechanical detection (MDT) was found in 29 survivors (63%), decreased

Paper	Study Design	Sample Size	Median Age at Diagnosis (years)	Median Age at Study (years)	Median Time Since Diagnosis /Treatment (years)	Number of Controls	Pain Outcomes Assessed	Pain Outcome Findings
								vibration detection (VDT) in 33 survivors (72%). Decreased cold detection (CDT) was found in 9 survivors (20%), and warm detection (WDT) in 6 survivors (13%).
Odame I.	Observational – Cross-Sectional	25	Mean 8.48	Mean 15.56	Mean 6.5	N/A	Health Utilities Index (HUI) – Pain item	Radiation therapy is associated with significant loss of bone mineral. Among these survivors, HRQL is less, pain is more severe and ambulation is

Paper	Study Design	Sample Size	Median Age at Diagnosis (years)	Median Age at Study (years)	Median Time Since Diagnosis /Treatment (years)	Number of Controls	Pain Outcomes Assessed	Pain Outcome Findings
								more restricted in those with low BMD scores.
Portwine et al.	Observational – Cohort	99	Mean 3.6	Not specified but 5–18years	Mean 4.36	N/A	Health Utilities Index (HUI2 and HUI3) – Pain item	Parents reported morbidity in sensation (52.5%), pain (30.3%), cognition (28%), and emotion (24.2%) and in hearing (38.4%), pain (30.3%), cognition (27.3%) and speech (23.3%) On the HUI3 pain was more often affected in high-risk neoblastoma in comparison to survivors of ALL, Wilms,

Paper	Study Design	Sample Size	Median Age at Diagnosis (years)	Median Age at Study (years)	Median Time Since Diagnosis /Treatment (years)	Number of Controls	Pain Outcomes Assessed	Pain Outcome Findings
								tumor. Mean pain scores were reported for NBL, NBL no HSCT, Wilms tumor, ALL and the general.
Van Dijk et al.	Observational – Cross Sectional	60	Mean 8.3	Mean 24.6	Mean 15.2	N/A	36-Item Short Form Health Survey (SF-36) – Bodily Pain scale	Patients treated before turning 12 years old reported significantly more pain compared to those treated after turning 12 years old.
Revel-Vilk et al.	Observational – Cross Sectional	51	Mean 6.5	Mean 10.5	Mean 2.3	N/A	Items created by the authors	Pain was reported by 5 (9.5%) patients, 2 of whom complained of limitation of function due to pain. Mild PTS was

Paper	Study Design	Sample Size	Median Age at Diagnosis (years)	Median Age at Study (years)	Median Time Since Diagnosis /Treatment (years)	Number of Controls	Pain Outcomes Assessed	Pain Outcome Findings
								present in 20 children (39%, 95% CI 26–54%). Pain was reported in five (9.5%, 95% CI 3.3–21.4%); two of these children complained of limitation of function due to pain. One patient scored 2 in Kuhle’s Pediatric PTS score for subjective symptoms of pain and arm swelling. Nineteen children scored 1 on Kuhle’s Pediatric PTS score for subjective

Paper	Study Design	Sample Size	Median Age at Diagnosis (years)	Median Age at Study (years)	Median Time Since Diagnosis /Treatment (years)	Number of Controls	Pain Outcomes Assessed	Pain Outcome Findings
								symptoms of pain.
Sadighi et al.	Observational – Cross Sectiona	162	Mean 3.9	Mean 15.7	Mean 10.2	N/A	Pediatric Migraine Disability Assessment Scale (PedMIDAS)/ 36-Item Short Form Health Survey (SF-36)	survivors had headache 39 (54%) females and 37 of 90 (41%) males. 61 (37.7%) developed headaches after diagnosis. The median time to diagnose headache is 5.4 years. Migraine headaches were diagnosed in 51 (31%) of survivors of whom 24 had also aura. Episodic tension-type headaches were

Paper	Study Design	Sample Size	Median Age at Diagnosis (years)	Median Age at Study (years)	Median Time Since Diagnosis /Treatment (years)	Number of Controls	Pain Outcomes Assessed	Pain Outcome Findings
								diagnosed in 49 (30%) survivors. In 76 survivors with headache, both migraine headaches and episodic tension-type headache. 13 (17%) reported a frequent need to interrupt activities during a headache, 36 patients (47%) occasionally interrupted activities during a headache, and 27 (36%) did not need to restrict physical activity during a

Paper	Study Design	Sample Size	Median Age at Diagnosis (years)	Median Age at Study (years)	Median Time Since Diagnosis /Treatment (years)	Number of Controls	Pain Outcomes Assessed	Pain Outcome Findings
								headache
Schultz et al.	Observational – Cross Sectional	180	Not specified, between 0-20	Not specified Between 8 to 30	Not Specified but 5-year survivor	N/A	Items created by the authors	Overall, slightly more than half (55%) of participants reported at least one chronic health Condition. Eight (5%) participants reported cancer-related pain. Survivors with more chronic health conditions or with cancer-related pain had diminished HRQL.
Schwartz et al.	Observational – Cross Sectional	156	Mean 19.9	Not specified Between 16 to 30	Not Specified but 5-year survivor	138 survivors	Health Knowledge Inventory	more health problems on survivors, such as

Paper	Study Design	Sample Size	Median Age at Diagnosis (years)	Median Age at Study (years)	Median Time Since Diagnosis /Treatment (years)	Number of Controls	Pain Outcomes Assessed	Pain Outcome Findings
								growth, hearing, thyroid, kidney, liver, immunological, heart, and fertility problems.
Williamson Lewis R.	Observational – Cross Sectional	579	Not specified Between 0 to 19	Not specified, Between 2 to 21	Not Specified but 1 year after treatment	N/A	Items created by the authors	dental problems (31.3%) the neurologic cluster was the most different from the full sample, comprising any chronic pain, back pain, and speech problems in young adults and back pain, abdominal pain, and dental

Paper	Study Design	Sample Size	Median Age at Diagnosis (years)	Median Age at Study (years)	Median Time Since Diagnosis /Treatment (years)	Number of Controls	Pain Outcomes Assessed	Pain Outcome Findings
								problems. The struc- ture of the neurologic cluster also differed greatly in males (67.2) and those ≥ 5 years old at diagnosis (68.2).

Table S3: Psychometric domains

Paper	Questionnaire	Psychometric Measures	Domain
Arpaci T. et al.	Items developed by authors	-	Physical or psychosocial problems and symptoms, Demographic, cancer and treatment, symptoms such as pain, fatigue, exercise, anorexia, nausea, insomnia, weight loss, food intolerance
Berg C. et al.	The Living Well Survey, the Adolescent Activity Card Sort, Participation Survey/ Mobility	+	Quality of Life, Activities of Daily Living, Functional Mobility, Life Participation
Bowers D.C.	Behavioral Risk Factor Surveillance System Survey Questionnaire, Items developed by authors	+	Quality of life
Brinkman T.M.	Behavior Problems Index	+	antisocial behavior, anxiousness/depression, headstrongness, hyperactivity, immature dependency, and peer conflict/social withdrawal.
Crom D.B.	European Organization for the Research and Treatment of Cancer (EORTC QLQ-C30) – Pain subscale	+	vision, hearing, cardiovascular function, endocrine status, gastrointestinal problems, hematological status, musculoskeletal integrity, neurological status, neurocognitive functioning, genitourinary function, and pubertal progression/reproductive status

Paper	Questionnaire	Psychometric Measures	Domain
Fenny D.	Health Utilities Index (HUI) – Pain item	+	physical function and mobility, cognition, sensation (hearing, speech, fertility and vision), pain, self care and emotion
Hsiao C.C	Intensity of treatment rating 2.0, Adverse Health Event Index (AHEI)	-	Quality of life, frequency and severity of adverse health events
Khan B.R.	Dizziness Handicap Inventory questionnaire; The Brief Fatigue Inventory; Migraine Disability Assessment Scale (MIDAS); Liverpool Seizure Severity Score; Common Terminology Criteria for Adverse Events v4.0 (CTCAE); Hanover Low Back Pain Disability Questionnaire; Vanderbilt Attention-Deficit Hyperactivity Disorder Parent Rating Scale; Scale for Assessment and Rating of Ataxia (SARA); The	+/-	dizziness on physical, functional, and emotional function, fatigue, migraine headaches, seizure, cranial nerve function and sensory, motor, and autonomic symptoms of neuropathy, pain, attention deficit, hyperactivity, ataxia, quality of life.

Paper	Questionnaire	Psychometric Measures	Domain
	Medical Outcome Survey Short Form-36 (SF36)		
Kranick S.M.	Chart review	-	Headache and strokes
Lieber S.	Reduced Pediatric-Modified Total Neuropathy Score (rPed-mTNS)/ QST	+	sensory, motor function, and autonomic symptom, pain
Odame I.	Health Utilities Index (HUI) – Pain item	+	Quality of Life, sensation, mobility, emotion, cognition, self-care, pain, and fertility, vision, hearing, speech, ambulation, dexterity, emotion, cognition, and pain.
Portwine et al.	Health Utilities Index (HUI2 and HUI3) – Pain item	+	Quality of Life, sensation, mobility, emotion, cognition, self-care, pain, and fertility, vision, hearing, speech, ambulation, dexterity, emotion, cognition, and pain.

Paper	Questionnaire	Psychometric Measures	Domain
Van Dijk et al.	40-item questionnaire that was developed for this study/36-Item Short Form Health Survey (SF-36)	-/+	General health, anxiety, depression, pain, somatization
Revel-Vilk et al.	Items created by the authors	-	Pain, PTS
Sadighi et al.	Pediatric Migraine Disability Assessment Scale (PedMIDAS)/ 36-Item Short Form Health Survey (SF-36)	+/+	Headache, Quality of Life, Pain
Schultz et al.	Items created by the authors, Childhood Cancer Survivor Study, SF-36	-/+	quality of life, medical conditions, socioeconomic status, and need for health care interventions, presence of chronic health conditions, education and employmen

Paper	Questionnaire	Psychometric Measures	Domain
Schwartz et al.	Health Knowledge Inventory	+	Fatigue, pain,
Williamson Lewis R.	Items created by the authors	-	Pain, depression, anxiety
